# Analysis of male specific region of the human Y chromosome sheds light on historical events in Nazi occupied eastern Poland

**DOI:** 10.1007/s00414-018-1943-0

**Published:** 2018-10-16

**Authors:** Marta Diepenbroek, Sandra Cytacka, Maria Szargut, Joanna Arciszewska, Grażyna Zielińska, Andrzej Ossowski

**Affiliations:** Department of Forensic Genetics, Pomeranian Medical University in Szczecin, Powstańców Wielkopolskich Street 72, Szczecin, Poland

**Keywords:** Y-STR, Y chromosome, Haplogroup, Mass grave, Exhumation

## Abstract

In Poland, during the World War II, almost 3 million people were killed during the Nazi occupation, and about 570,000 during the Soviet occupation. Furthermore, historians have estimated that after the World War II at least 30,000 people were killed during the Stalinist regime in Poland (1944–1956). The exact number is unknown, because both executions and burials were kept secret. Thousands of people just vanished. As a response to those events, forensic scientists from the Pomeranian Medical University in Szczecin in cooperation with historians from the Institute of National Remembrance started the project of the Polish Genetic Database of Victims of Totalitarianism, which aim is to identify victims killed in the years 1939–1956. Several exhumations were done under the project, with the biggest one done in Białystok. According to the information gathered by local historians, a detention centre in Białystok was the place of the secret burials in late 1940s and 1950s. Surprisingly, except few graves from the post-war period, most of the burials found in Białystok indicated that majority the victims were probably local civilians who died during the Nazi occupation. Unfortunately, data concerning what happened in the detention ward during that period of time is not very detailed. What was known is that people who got incarcerated were “political prisoners” what, according to Nazi politics, was based on their nationality, religion and activity against the Third Reich. The aim of this research was to test genetically the remains found in Białystok to determine their possible ethnic background, in order to shed new light on the victims and what happened in the Białystok detention centre during the Nazi occupation. The analysis of male specific region of the human Y chromosome shows that including phylogenetic analysis into the complex process led by the Polish Genetic Database of Victims of Totalitarianism may help with the final identification of hundreds of anonymous victims.

## Introduction

In World War II, nearly 3 million and about 570,000 people were killed during the Nazi and the Soviet occupation of Poland, respectively [1]. Furthermore, historians have estimated that after World War II, at least 30,000 people were killed during the Stalinist regime in Poland (1944–1956) [2]. The exact number is unknown, because both executions and burials were kept secret. Thousands of people just vanished [2].

In 2012, forensic scientists from the Pomeranian Medical University in Szczecin started, in cooperation with historians from the Institute of National Remembrance, the project called “The Polish Genetic Database of Victims of Totalitarianism” [3]. It was created as a tool for the identification of communist terror victims, killed in the years 1944–1956. The project is a response to historical events.

The biggest exhumation work done under this project happened in the eastern part of Poland, in Białystok, the capital of the Podlaskie province. According to information gathered by local historians, a detention centre in Białystok city centre was the place of secret burials of communist victims [4]. The main aim of the project was to identify the remains found in Białystok. Based on the initial hypothesis that the victims were killed in the years 1944–1956, the gathered reference material applied mostly to people who disappeared after World War II. But surprisingly, except for a few graves from the post-war period, most of the burials found in Białystok indicated that the majority the victims were probably local civilians who died during the Nazi occupation. Due to that, new families had had to be found—also the ones who lost their relatives during World War II. Unfortunately, data concerning what happened in the detention ward during that period of time is not very detailed, as the Gestapo destroyed most of their archives before leaving Białystok. It was, therefore, hard to create a list with the names of possible victims. What is known is that people who got incarcerated were both Polish underground activists and accidental civilians from the whole province. Inmates were called “political prisoners” what, according to Nazi politics, was based on their nationality, religion, and activity against the Third Reich.

## Aim

By molecular-genetically testing of the human remains found on site, this research aimed at shedding new light on the victims and gaining insights on what happened in the Białystok detention centre during the Nazi occupation. For this purpose, Y-chromosomal STR markers were analysed on the remains of 100 male victims.

## Materials and methods

### Exhumation

The detention centre in Białystok was built in 1906. Before World War II, the detainees were mostly convicted on criminal charges. But starting in 1939, the detention centre became a place to keep not only criminals. Between 1939 and 1941, it stayed under authority of NKVD, and when the Nazi occupation started in 1941, it went under the control of the Gestapo until 1944. From 1944, the detention centre was subjected to the Polish Ministry of Public Security and got filled with people from the political opposition.

The place has changed over the years. At the turn of the 1960s, the garden of the detention ward was changed into an economic space, which was still there when the exhumation work started. The research group believed they would find hidden remains of communism opponents killed by the government just after the war. During the exhumation, a few remains of people shot in the back of the head were found [5]. They were carrying personal belongings, which indicated that the victims indeed were underground activists. However, most of the graves differed by being mass graves with women, men, children, and seniors inside. Most of the remains showed no signs of trauma on the bones and the remains were often covered with calcium and potassium permanganate. The artefacts found in many graves were identified as basic personal belongings, indicating that the victims were most probably local civilians who died during the Nazi occupation. One of the graves had even stronger evidence—it was an execution grave, with 24 people killed by a Nazi firing squad [5].

The field work in Białystok took 2 years and consisted of six stages. Finally, over 300 remains in 66 graves were found. Of these remains, 177 were attributed to men, 61 to women, and 141 came from children.

### Biological material

DNA material for analysis was sampled from all 177 male remains found in the former gardens of the detention centre in Białystok. The individuals were chosen from among all the exhumed remains based on the anthropological assessment. The victims were 20–60 years old, with an average age of about 40 years. This means, according to the historical data, that they were born at the beginning of the twentieth century and lived in the region of Podlaskie province.

A team of “The Polish Genetic Database of Victims of Totalitarianism” [3] conducted the exhumations. Forensic anthropologists, supervised by geneticists, collected healthy molars from each skeleton. Most of the remains were found in mass graves and the collected biological material was classified as being highly degraded.

### Preparation of teeth for extraction of DNA

The teeth were mechanically cleaned from surface deposits using a special tool from Proxxon and sterile diamond grids (Proxxon) [3]. Next, they were chemically cleaned by a 15-min wash in 15% sodium hypochlorite solution [3] and rinsed with distilled water. After that, the teeth were sterilised by UV-C irradiation for 30 min and air-dried in a laminar flow chamber.

The pre-treated teeth were placed in a cryogenic laboratory grinder 6870 Freezer/Mill® by Spex SamplePrep, chilled with liquid nitrogen, and then milled to fine powder.

### DNA extraction

About 0.05 g of tooth powder were used for extraction done with the PrepFiler® BTA Forensic DNA Extraction Kit (ThermoFisher Scientific) according to the manufacturer’s instructions. Each sample was extracted at least two times.

### DNA quantification

The Quantifiler Trio DNA Quantification Kit (TFS) was used to assess the DNA concentrations of the extracts and to identify samples being potentially compromised by unintentionally co-extracted PCR inhibitors.

### DNA amplification and electrophoretic separation

DNA was amplified using the Yfiler Plus PCR Amplification Kit (TFS) according to the manufacturer’s protocol. DNA input for the 25-μl reactions was within the optimum range recommended by the manufacturer and 30 thermal cycles were applied on an Applied Biosystems Veriti Thermal Cycler (TFS).

Electrophoretic sizing of the PCR products was performed on a 3500 Genetic Analyzer using 600 LIZ™ as internal size standard and the GeneMapper® ID-X software for data processing (both: TFS).

### Y-haplogroup estimation

Y-haplogroup estimation was done using Nevgen [6]. This online tool uses Bayesian-Allele-Frequency to estimate to which haplogroup a Y-STR haplotype belongs. For the estimations, 23 markers were used: DYS576, DYS389I, DYS635, DYS389II, DYS460, DYS458, DYS19, YGATAH4, DYS448, DYS391, DYS456, DYS390, DYS438, DYS392, DYS570, DYS437, DYS385a/b, DYS449, DYS393, DYS439, DYS481, and DYS533. The predictor model was based on automatic selection, and the final estimates were the haplogroups suggested by Nevgen with the highest probability.

### Biogeographical background of Y-STR profiles

In an attempt to shed light on the spatial distribution of the Y-STR profiles found in this research, we queried the Y-HRD database, which holds worldwide information on Y-chromosomal variation at the level of Y-STR haplotypes and corresponding haplogroups. In its current version (release 57), the Y-HRD holds five times more Yfiler than Yfiler Plus profiles. The 165,259 Yfiler haplotypes come from 118 national databases and 4683 of them are from Poland. For database queries, we, therefore, trimmed the 27-locus Yfiler Plus profiles to the 17-locus Yfiler haplotypes. Furthermore, to achieve reliable results, only full or 16-locus Yfiler profiles were considered for addressing the biogeographical background of our Y-STR data.

## Results

From the 177 studied individuals described as males by the anthropological assessment, the Y-chromosomal analysis failed for 77 (due to the high degradation of bone material or incorrect sex estimation). For the remaining 100 individuals, satisfying Y-STR data was obtained. These samples were used for further analyses. Seventy samples yielded data for ≥ 20 Y-STRs, whereas allele-calls for 16–19 loci were obtained for the other 30 specimens. The haplotypes presented in Table 1 represent consensus profiles based on multiple amplifications.

**Table 1:** The haplotypes based on 23 studied Y-STRs

Sample	DYS576	DYS389I	DYS635	DYS389II	DYS460	DYS458	DYS19	YGATAH4	DYS448	DYS391	DYS456	DYS390	DYS438	DYS392	DYS570	DYS437	DYS385a/b	DYS449	DYS393	DYS439	DYS481	DYS533
223	16	12	21	28	11	15	15	11	20	11	14	22	10	n/a	19	16	14	n/a	13	11	25	11
225	16	12	21	29	10	15	14	12	20	10	14	23	10	11	20	16	13,14	28	14	11	25	n/a
228	18	14	20	31	9	17	14	10	20	10	16	23	9	11	17	14	13,16	32	12	11	n/a	n/a
232	16	14	21	29	11	17	14	12	n/a	11	14	23	10	14	18	14	11	33	14	10	20	n/a
234	17	13	23	29	11	16	16	11	n/a	10	17	24	11	11	19	14	11,14	n/a	13	12	26	n/a
233	18	13	24	n/a	10	16	17	11	n/a	n/a	15	23	10	11	18	16	13,15	n/a	13	13	28	n/a
236	19	12	21	29	10	16	13	9	n/a	n/a	15	23	10	11	19	14	16,18	32	13	12	27	n/a
240	18	13	25	31	10	16	16	11	19	n/a	15	24	10	n/a	18	15	14,15	31	n/a	13	31	n/a
242	16	14	23	31	n/a	16	17	13	20	11	15	25	11	11	21	14	12,14	n/a	13	11	23	12
243	16	14	23	30	11	17	15	12	n/a	11	12	23	10	14	19	14	11,13	n/a	14	10	20	11
244	19	13	23	30	11	18	16	12	20	10	15	24	11	11	19	14	10,14	32	13	10	26	12
245	18	13	23	29	10	16	16	12	20	n/a	17	25	11	11	n/a	n/a	15	n/a	13	11	22	12
247	17	13	24	30	11	15	16	12	20	11	16	26	11	11	19	14	11,14	32	13	10	24	12
248	17	14	21	30	10	14	15	12	21	9	16	23	9	11	18	14	13,16	31	12	11	23	12
251	18	14	23	33	10	17	16	11	20	10	15	23	10	11	19	15	14,15	32	13	13	29	13
252	18	13	23	29	10	16	16	12	20	10	17	25	11	n/a	16	14	11,14	n/a	13	12	28	12
253	18	13	24	32	10	16	16	11	20	n/a	15	24	10	11	n/a	15	14,15	n/a	13	13	29	n/a
254	17	13	22	29	11	16	15	12	19	12	14	23	10	14	19	14	11,13	28	13	10	20	n/a
255	16	12	23	28	11	17	14	12	19	n/a	16	24	12	13	16	15	11,14	29	13	13	21	11
258	15	14	22	30	10	17	15	12	19	10	13	23	10	15	19	n/a	11,13	28	14	10	20	12
259	17	13	20	28	9	15	13	11	21	10	15	23	10	12	18	15	14,15	26	13	11	23	10
260	18	13	23	32	n/a	17	16	11	20	11	15	24	10	11	18	14	14	32	13	12	30	12
261	15	12	22	29	11	17	15	12	20	10	15	23	10	11	17	16	13,14	n/a	14	11	21	n/a
264	20	14	23	n/a	11	16	17	13	20	10	16	25	12	11	19	14	10,14	31	14	11	n/a	12
273	19	13	23	29	10	16	16	12	20	n/a	16	26	11	11	19	14	11,14	32	13	11	26	12
276	18	13	23	n/a	11	17	16	12	n/a	10	16	25	11	15	17	14	10,15	34	13	10	25	12
277	19	13	23	29	10	16	16	12	20	n/a	16	26	11	11	19	14	11,14	32	13	11	26	12
278	18	13	23	30	11	16	16	12	21	11	16	25	10	11	19	14	11,14	31	13	10	25	10
279	18	13	23	32	10	17	16	11	20	11	15	24	10	11	18	15	15	32	13	13	31	13
280	20	13	23	29	12	16	17	13	20	10	18	25	11	11	18	14	11,14	31	13	11	23	12
282	17	13	20	31	10	18.2	14	11	20	10	14	23	10	11	17	14	13,18	26	12	11	25	11
284	18	14	23	30	11	15	16	13	20	11	17	25	11	11	18	14	11,14	32	13	10	23	12
285	18	13	25	31	11	18	15	12	20	11	14	25	11	11	19	14	11,14	34	13	10	24	13
288	16	14	24	31	10	18	14	11	19	11	15	19	10	13	17	14	13	n/a	13	13	24	n/a
291	21	13	23	30	12	15	16	12	19	n/a	16	25	11	n/a	18	14	13,14	31	13	10	23	12
297	17	13	23	29	11	17	14	12	19	n/a	16	24	12	13	n/a	15	11	30	13	12	n/a	n/a
298	18	13	23	29	11	17	15	12	20	10	16	25	15	11	19	14	11,14	31	13	11	24	n/a
299	16	13	23	29	11	17	15	12	19	n/a	13	23	10	14	17	14	11,13	28	14	10	20	11
306	19	14	23	30	11	17	14	12	18	n/a	15	24	12	n/a	17	15	11,14	29	13	13	n/a	12
307	19	13	23	29	11	15	14	12	19	n/a	14	24	12	13	19	15	11,14	31	12	11	21	11
312	19	14	20	30	11	17	15	11	19	10	16	22	9	11	16	14	15,17	31	12	11	23	11
314	19	14	20	30	11	19	15	n/a	19	8	15	22	9	11	17	15	15,17	31	12	11	23	12
315	15	12	21	28	10	16	15	n/a	n/a	10	14	22	10	11	18	16	13	28	13	n/a	25	n/a
318	18	11	20	27	11	18	15	12	19	n/a	15	22	9	11	16	14	16	32	12	11	23	n/a
322	15	13	24	29	10	18	15	12	19	n/a	15	23	10	n/a	22	14	11,14	28	14	10	20	11
329	16	12	21	28	10	16	14	11	20	n/a	14	22	10	12	20	16	14	29	13	11	27	11
332	17	13	22	n/a	10	15	15	10	20	10	14	23	10	n/a	20	14	15	n/a	15	11	27	11
337	16	12	21	28	11	16	15	11	20	13	14	22	10	12	n/a	16	14	n/a	13	11	n/a	n/a
341	18	14	20	n/a	11	18	15	12	n/a	n/a	15	22	9	11	16	14	15,17	31	12	11	23	12
343	16	12	n/a	28	10	16	n/a	11	n/a	11	14	22	10	12	20	16	14	n/a	13	11	27	n/a
358	17	13	23	29	10	16	14	12	19	11	16	25	12	13	17	15	11,14	29	13	12	23	n/a
360	16	12	21	28	11	15	15	11	20	n/a	14	22	10	12	19	16	14	28	13	11	25	n/a
366	18	14	23	31	10	17	14	11	18	11	15	24	n/a	13	17	14	11,14	30	13	12	24	12
371	18	13	23	30	10	15	17	12	22	11	17	25	11	11	18	14	11,14	32	13	10	23	12
373	16	14	21	29	11	17	14	12	19	11	14	23	10	14	18	14	11,13	33	14	10	20	11
377	20	13	23	30	9	15	n/a	12	20	10	16	25	n/a	11	20	14	16,18	n/a	13	n/a	22	12
379	18	12	23	29	12	15	16	13	21	10	16	25	11	11	18	14	11,14	29	13	10	23	12
380	18	n/a	23	n/a	10	16	16	n/a	20	10	17	n/a	11	n/a	19	14	11,14	32	12	n/a	25	13
381	17	13	23	29	11	16	15	12	20	10	17	25	11	11	19	14	11,15	n/a	13	11	25	12
384	19	13	23	30	11	16	17	13	20	11	15	23	11	11	20	14	12,13	32	13	12	23	12
386	17	13	24	30	n/a	15	16	13	20	11	15	25	11	11	20	14	11,15	n/a	13	10	23	12
387	18	13	24	n/a	11	15	16	13	21	n/a	15	24	11	n/a	18	14	11,14	31	13	10	23	n/a
404	16	n/a	24	n/a	11	17	14	n/a	19	n/a	15	23	12	13	17	15	11,14	n/a	13	13	22	12
410	18	14	23	32	11	n/a	14	n/a	18	10	17	n/a	12	13	17	14	11,14	n/a	13	12	22	n/a
413	19	13	22	29	11	16	16	11	20	10	14	22	10	13	20	14	16,17	27	13	11	26	13
421	18	13	23	29	11	16	17	12	20	10	17	25	11	11	19	14	11,14	33	13	11	25	12
424	19	13	23	28	11	15	14	12	19	10	16	24	12	14	17	14	11,14	29	12	13	n/a	n/a
426	12	13	21	31	10	16	13	11	20	9	15	25	10	11	16	14	17,18	28	14	12	25	11
427	17	13	23	29	10	16	16	11	20	10	17	24	11	11	19	14	11,14	31	13	11	25	12
428	17	12	23	n/a	10	17	14	11	19	n/a	15	23	12	13	17	15	11,14	30	13	11	22	12
432	18	13	23	30	11	16	n/a	12	20	10	16	25	11	11	21	14	10,14	31	13	11	25	12
434	17	13	23	30	11	15	16	13	20	10	16	25	11	11	20	14	11,13	33	13	11	23	n/a
436	17	12	24	28	10	15	n/a	11	20	n/a	14	23	10	11	21	16	14	n/a	13	12	26	n/a
546	19	13	23	30	11	16	16	12	19	10	16	24	11	11	19	14	10	31	13	10	n/a	n/a
547	18	12	23	28	10	14	14	11	20	n/a	15	23	10	n/a	20	15	13,15	29	13	11	25	11
548	15	12	23	28	10	15	14	11	20	n/a	15	22	10	11	19	16	12,14	31	13	10	25	11
549	16	13	23	29	11	15	16	11	20	13	15	24	11	11	18	14	11,14	n/a	13	10	23	12
550	18	14	23	30	11	16	15	12	20	10	17	25	11	11	19	14	11,14	n/a	13	11	25	12
551	18	13	23	30	10	17	16	11	20	9	15	24	10	11	18	15	14,15	31	14	13	30	12
553	18	13	22	31	n/a	16	16	11	20	11	15	24	10	11	18	14	14,15	31	13	12	31	n/a
554	18	13	23	29	11	16	16	12	20	10	17	25	11	11	19	14	11,14	32	13	11	25	12
555	15	n/a	24	n/a	10	19	16	12	n/a	10	15	23	10	14	22	14	11,14	28	14	n/a	20	11
557	17	13	23	30	12	14	15	13	20	n/a	15	25	11	11	18	14	11,15	31	13	11	23	n/a
559	18	12	23	30	n/a	16	15	12	20	11	15	26	11	11	19	14	11,14	33	13	11	24	n/a
561	19	14	22	30	n/a	19.2	14	10	20	10	14	23	10	11	18	14	13,18	26	12	11	28	12
566	18	14	23	31	10	17	14	11	18	11	15	24	n/a	13	17	14	11,14	30	13	12	24	12
571	16	14	21	30	n/a	15	15	11	20	9	15	23	10	13	18	14	15	30	14	11	28	n/a
574	15	14	21	30	10	17	15	12	19	11	13	23	n/a	15	18	14	11	28	14	11	20	n/a
580	18	14	20	31	9	17	14	10	20	10	16	23	9	11	17	14	13,16	32	12	11	23	14
592	16	12	21	28	11	15	15	11	20	11	14	22	10	12	19	16	14	29	13	11	25	n/a
595	19	14	20	30	n/a	18	15	12	19	10	15	22	9	11	n/a	14	15,17	n/a	12	11	n/a	n/a
601	16	12	21	28	10	16	14	11	20	n/a	14	22	10	12	19	16	14	29	13	11	27	n/a
603	17	13	23	30	n/a	15	17	13	20	11	16	25	11	11	n/a	14	11,13	33	13	11	23	n/a
605	17	13	23	29	10	16	14	12	19	n/a	16	25	12	13	17	15	11,14	29	13	12	23	12
614	16	14	23	33	12	15	17	n/a	21	n/a	17	24	11	11	19	14	11,14	32	13	11	23	n/a
615	15	13	23	29	11	17	14	12	19	n/a	16	25	12	13	17	15	11,15	29	13	12	22	n/a
635	16	12	21	28	10	16	14	11	20	n/a	14	22	10	12	20	16	14	29	13	11	n/a	n/a
638	18	11	20	n/a	11	18	15	12	n/a	n/a	15	22	n/a	11	16	14	15,17	n/a	12	11	23	n/a
640	19	13	21	29	11	16	16	12	20	n/a	17	25	11	11	n/a	14	11,13	n/a	9	12	n/a	n/a
641	19	12	21	28	10	16	14	11	21	10	14	23	10	11	n/a	16	13,14	n/a	13	11	n/a	n/a

*n/a* not amplified

For 67 individuals, full Yfiler haplotypes (or 1 marker missing) were obtained and queried against the Y-HRD database. Samples not producing direct matches were subjected to one-step neighbour analysis. Results are summarised in Tables 2 and 3.

**Table 2:** The results of Y-HRD analysis for the samples showing matches in the YFiler database

Sample	Haplogroup estimation (based on Nevgen)	Y-HRD matches (based on 165,259 haplotypes)	Matches in Poland (based on 4683 haplotypes)	Matches worldwide (n/number of haplotypes)
244	R1a	1	0	Germany (1/4786)
252	R1a	27	17	Germany (3/4786)
				Brazil (2/8043)
				Austria (1/1516)
				Bosnia and Herzegovina (1/300)
				Latvia (1/139)
				Lithuania (1/532)
				Russia (1/2139)
255	R1a	3	0	Belgium (1/1168)
				Ireland (1/872)
				Netherlands (1/2345)
273	R1a	3	0	China (1/59655)
				Germany (1/4786)
				Lithuania (1/532)
277	R1a	3	0	China (1/59655)
				Germany (1/4786)
				Lithuania (1/532)
279	I2	14	3	Austria (1/1516)
				Bulgaria (1/318)
				Croatia (1/1339)
				Germany (1/4786)
				Greece (1/595)
				Hungary (1/937)
				Latvia (1/139)
				Russia (1/2139)
				Serbia (3/603)
282	J-P58	2	0	Saudi Arabia (1/597)
				Turkey (1/1460)
312	H-M82	1	0	Iran (1/1688)
358	R1b	6	0	Australia (1/2256)
				France (1/557)
				Norway (2/1574)
				Spain (1/6955)
				USA (1/6825)
366	R1b	14	0	Argentina (4/2901)
				Chile (3/977)
				USA (3/6825)
				Spain (2/6955)
				Brazil (1/8043)
				Ecuador (1/1025)
373	N1c	1	0	Russia (1/2139)
381	R1a	1	1	0
386	R1a	27	6	Russia (11/2139)
				China (1/59655)
				Estonia (1/125)
				Germany (2/4786)
				Latvia (1/139)
				Lithuania (5/532)
421	R1a	7	4	Russia (2/2139)
				Spain (1/6955)
426	E-M123	1	0	UK (1/3930)
427	R1a	1	1	0
432	R1a	28	10	Croatia (4/1339)
				Germany (3/4786)
				Austria (2/1516)
				Russia (2/2139)
				Czech Republic (1/114)
				Italy (1/3366)
				Latvia (1/139)
				Serbia (1/603)
				Slovenia (1/305)
				Turkey (1/1460)
				UK (1/3930)
548	I1	1	1	0
550	R1a	1	1	0
554	R1a	93	34	Russia (17/2139)
				Germany (11/4686)
				Serbia (5/603)
				Australia (3/2256)
				Hungary (3/937)
				Macedonia (3/515)
				Estonia (2/125)
				Latvia (2/139)
				Ukraine (2/215)
				Austria (1/1516)
				Bosnia and Herzegovina (1/300)
				Brazil (1/8043)
				China (1/59655)
				Croatia (1/1339)
				Finland (1/431)
				Greece (1/595)
				Lithuania (1/532)
				Slovakia (1/256)
				Spain (1/6955)
				Turkey (1/1460)
557	R1a	1	1	0
566	R1b	14	0	Argentina (4/2901)
				Chile (3/977)
				USA (3/6825)
				Spain (2/6955)
				Brazil (1/8043)
				Ecuador (1/1025)
592	I1	3	2	Germany (1/4786)
595	H-M82	139	1	Hungary (Romani) (30/937)
				Romania (Romani) (29/373)
				Bulgaria (Romani) (19/318)
				Greece (Romani) (14/595)
				Slovakia (11/256)
				Croatia (9/1339)
				Turkey (7/1460)
				Ukraine (Romani) (5/215)
				Albania (2/322)
				Germany (2/4786)
				Serbia (2/603)
				UK (2/3930)
				USA (European) (2/3930)
				India (Tami) (1/4169)
				Ireland (1/872)
				Latvia (1/139)
				Spain (Romani) (1/6955)
				Switzerland (1/893)
603	R1a	9	3	Austria (2/1516)
				Albania (1/322)
				Macedonia (1/515)
				Russia (1/2139)
				Serbia (1/603)
605	R1a	10	0	Spain (3/6955)
				Norway (2/1574)
				Australia (1/2256)
				Brazil (1/8043)
				France (1/557)
				Ireland (1/872)
				USA (1/6825)
615	R1a	7	0	Ireland (2/872)
				UK (2/3930)
				Norway (1/1574)
				Spain (1/6955)

**Table 3:** The results of Y-HRD analysis for the samples with no matches in the YFiler database

Sample	Haplogroup estimation (based on Nevgen)	One-step neighbours (base on 165,259 haplotypes)	Neighbours worldwide (n/number of haplotypes)
225	I1	0	0
228	J-Z7671	2	Poland (1/4683)
			Spain (1/6955)
234	R1a	5	Poland (2/4683)
			Germany (1/4786)
			Norway (1/1574)
			Serbia (1/603)
242	R1a	0	0
243	N1c	4	Lithuania (2/532)
			Germany (1/4786)
			Hungary (1/937)
247	R1a	9	Poland (4/4683)
			Russia (2/2139)
			Australia (1/2256)
			Belgium (1/1168)
			Norway (1/1574)
248	J-Z387	5	Brazil (5/8043)
			Italy (1/3366)
			Netherlands (1/2345)
			Spain (1/6955)
251	I2	0	0
253	I2	9	Poland (3/4683)
			Croatia (2/1339)
			Cyrpus (1/724)
			Germany (1/4786)
			Hungary (1/937)
			Russia (1/2139)
254	N1c	0	0
258	N1c	5	Lithuania (4/532)
			Russia (1/2139)
259	G-M342	0	0
260	I2	17	Russia (5/2139)
			Poland (3/4683)
			Albania (2/322)
			Croatia (2/1339)
			Argentina (1/2901)
			Italy (1/3366)
			Lithuania (1/532)
			Slovakia (1/256)
			USA (1/6825)
261	G-U1	1	China (1/59655)
264	R1a	0	0
278	R1a	18	China (17/59655)
			Germany (1/4786)
280	R1a	0	0
284	R1a	6	Lithuania (2/532)
			Poland (2/4683)
			Norway (1/1574)
			Russia (1/2139)
285	R1a	0	0
288	R1b	1	China (1/59655)
298	R1a	0	0
299	N1c	8	Lithuania (2/532)
			Finland (1/431)
			Germany (1/4786)
			Hungary (1/937)
			Latvia (1/139)
			Poland (1/4683)
			Russia (1/2139)
307	R1b	2	Brazil (1/8043)
			Germany (1/4786)
337	I1	0	0
371	R1a	0	0
379	R1a	0	0
384	R1a	0	0
413	I2	1	Netherlands (1/2345)
424	R1a	2	Mexico (1/2382)
			Spain (1/6955)
434	R1a	15	Austria (2/1516)
			Germany (2/4786)
			Italy (2/3366)
			Lithuania (2/532)
			Russia (2/2139)
			Spain (2/6955)
			China (1/59655)
			Poland (1/4683)
			Ukraine (1/215)
546	R1a	9	Poland (6/4683)
			Estonia (1/125)
			Germany (1/4786)
			UK (1/3930)
549	R1a	0	0
551	I2	2	Cyprus (1/724)
			Russia (1/2139)
553	I2	2	Lithuania (2/532)
559	R1a	0	0
561	J-P58	0	0
571	I2	0	0
580	J-Z7671	2	Poland (1/4683)
			Spain (1/6955)
640	R1a	0	0
641	I1	20	Germany (4/4786)
			Netherlands (4/2345)
			Austria (2/1516)
			Poland (2/4683)
			Spain (2/6955)
			Angola (1/71)
			Argentina (1/2901)
			Australia (1/2256)
			Slovenia (1/305)
			Sweden (1/302)
			USA (1/6825)

Among 27 haplotypes producing direct matches in Y-HRD database, 14 showed matches among Polish database. Over 70% (ten samples) of those were estimated by Nevgen as R1a haplogroup, two haplotypes as I1 haplogroup, one as I2 and 1 as H-M82. For the remaining 40 haplotypes with no direct matches, one-step neighbour analysis produced matches in Poland for 11 haplotypes.

**Fig. 1:**
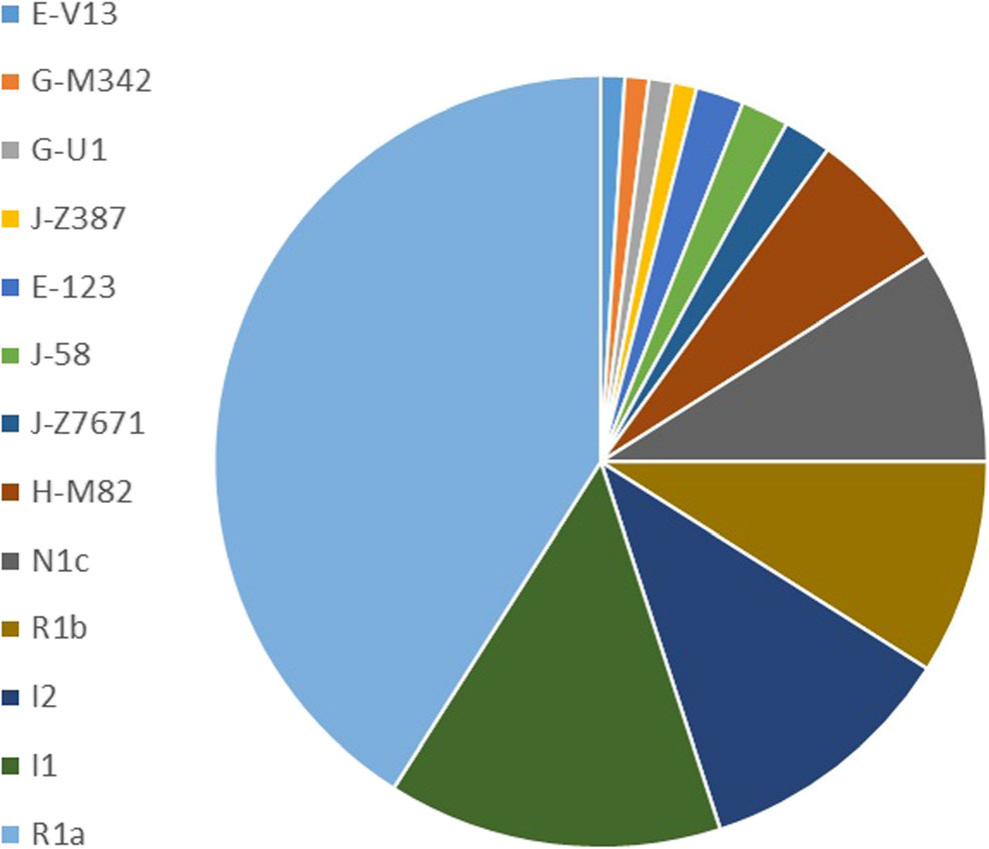
Nevgen haplogroup estimates

A subset of up to 23 Y-STRs was used for Nevgen haplogroup estimation on the 100 samples amplifying at least at 16 of the 27 Yfiler Plus loci. For 95 individuals, the probability of the estimation was nominally 100%. For the remaining five individuals, it ranged between 60% (haplogroups estimated as J-Z7671, E-M123 and G-M342) and 80% (G-U1 and J-Z7671). Results are presented in Fig. 1.

**Fig. 2:**
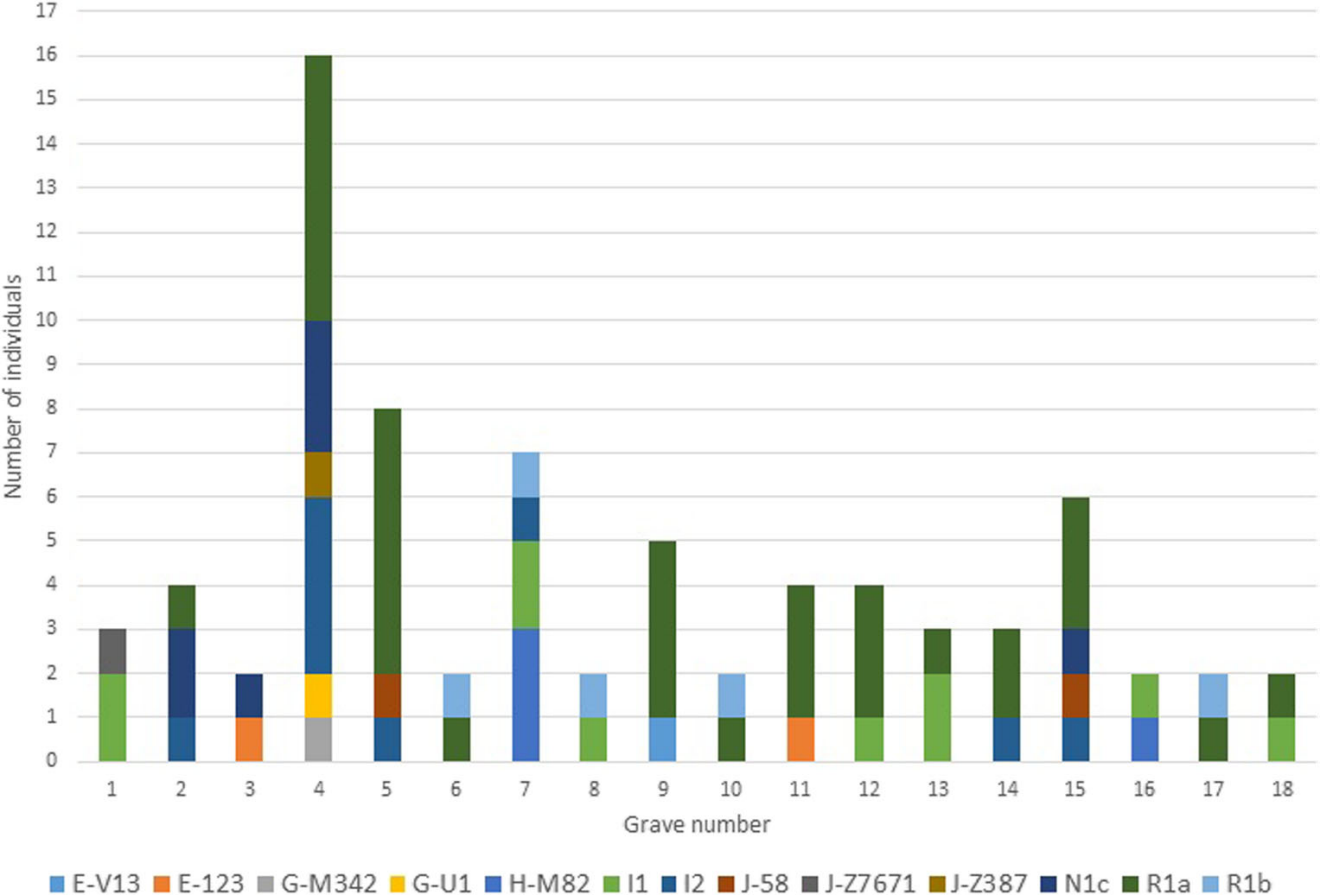
The distribution of estimated haplogroups among studied mass graves

Almost all of the individuals were found in mass graves of men, women, and children. In case of 18 graves, more than one male individual was analysed. The distribution of estimated haplogroups in the studied mass graves is presented in Fig. 2.

## Discussion

Analysis of markers on the MSY (the male specific region of the human Y chromosome) [7], facilitates reconstruction of paternal lineages. The MSY is passed down clonally and contains a plethora of single nucleotide variant markers as well as short tandem repeat loci, making it the largest pool of human genetic markers that are inherited in the form of a single haplotype. Mutational events in the germline result in groups of individuals carrying similar Y-chromosomal haplotypes, which in turn can be compiled into specific haplogroups. Thanks to that, it is possible to reconstruct the genealogical tree of humanity and to retrace historical migrations of male lineages, shedding some light into their possible ethnic background.

In our study, we analysed 23 Y-STR loci on the remains of 100 men exhumed at the detention centre in Białystok. In the light of historical data, those people lived in Podlaskie province and were killed in the ward between 1939 and 1956. Due to lacking archives, data about their identity was not very detailed. Gathered historical records suggest that the Bialystok detention centre was not a place of mass ethnic cleansing and that people who got incarcerated were both Polish underground activists and accidental civilians from the whole province.

On basis of up to 23 Y-STRs, the main Y-chromosomal haplogroup estimated for the studied individuals was R (50%, Fig. 1), which is known to be found among around half of the European populations [8, 9]. Most of the studied individuals (41%) were suggested as R1a, often called the “Slavic” haplogroup. Its high frequency in Eastern Europe was confirmed by different research groups [10–14]. Haplogroup R1b is known to be more prevalent for Western Europe [11, 15]. According to the study by Battaglia et al. [16], haplogroup R1a was found among Polish samples at a frequency around 56% and haplogroup R1b around 18%. Other authors [11] reported similar observations by mentioning haplogroups R as the most common in Poland and Podlaskie province. The following most frequent haplogroups were I (25%) and N (9%, Fig. 1), which are known to be common in European populations including Poland [11, 16].

Pepiński et al. [12] also studied male samples from Podlaskie. On basis of 186 haplotypes comprising 12 Y-STRs, these authors found no statistically significant discrepancy between the population of Podlaskie and other Polish populations.

Three of the studied individuals were assigned to haplogroup E, one being suggested as E-V13 and two as E-M123 (Fig. 1). Battaglia and colleagues [16] did not observe haplogroup E-M123 in the Polish sample although they reported it for other populations analysed in the very same paper [16]. This finding is in line with data published by Cruciani et al. [17] and Semino et al. [18], which also did not observe E-M123 Y chromosomes among Polish samples [17, 18]. Notably, some studies show that E-M123 is the most common E sub-haplogroup found among Ashkenazi Jews [19, 20].

Five of our studied individuals were placed by Nevgen within haplogroup J, which was found in Polish population samples by different studies [11, 16, 18]. Battaglia et al. [16] observed the J-M241 subclade, which did not occur among our samples. Studied individuals were estimated as J-Z387 and J-Z7671, both being J2a branches. Furthermore, Battaglia et al. [16] also reported the presence of J1 Y chromosomes (J-M267), a finding not mentioned by the other studies [11, 18]. Our N individuals from J1 were assigned to J-P58 (Fig. 1), which is called the “Semitic” branch. According to various research groups, this particular haplogroup is found almost exclusively among Ashkenazi Jews [19, 20].

Among the studied remains, 6% were estimated as H-M82. Pamjav et al. [21] studied a group of Roma people from Hungary and found out that H-M82 is the most frequent haplogroup among them. This discovery was confirmed by another publication on Romani samples [22]. However, the occurrence of haplogroup H-M82 Y chromosomes has not been reported for the general European population [23]. This applies to previously published papers including Polish samples [11, 16], too.

For our sample, the collective haplogroup G (G-M342 and G-U1) was estimated at the lowest frequency. In line with that, it was not mentioned by previous papers including Polish samples [11, 16]. Hammer and Behar reported haplogroup G as being rather frequent among Ashkenazi Jews and that G1 branch (G-M342 falls into that) is found within European populations with rather low frequencies [19, 20].

## Conclusions

The preliminary results presented in this paper shed some light on the possible ethnic background of the remains exhumed in Białystok. Most of the studied males (over 80%) were suggested as of European origin and represented haplogroups typical for Polish population. Nevertheless, some of the individuals got assigned to Y-chromosomal haplogroups known for being very rare in Europe. They might have belonged to ethnic minorities (Jews and Roma) being present in the Podlaskie province before World War II [24]. The available archives are uncertain about what had happened in the detention centre during the Nazi occupation. The distribution of the haplogroups among the studied mass graves suggests that the victims were buried all together irrespectively of their sex, age or ethnicity. Genetic results show that none of the found burials was dedicated only to one ethnic minority. Thus, our preliminary data rather suggest that the garden of the detention ward in Białystok was not used to hide the bodies of victims of mass ethnic cleansing but that the victims were local civilians representing multiple ethnic groups living in Podlaskie in the 1940s. The here presented data on Y-STRs adds to the scarce body of information that is available on the victims found in the Białystok detention centre. Including phylogenetic analysis into the complex process led by the Polish Genetic Database of Victims of Totalitarianism may help with the final identification of hundreds of anonymous victims.
